# Transmission risks of Omicron BA.5 following inactivated COVID-19 vaccines among children and adolescents in China

**DOI:** 10.1038/s43856-024-00521-y

**Published:** 2024-05-18

**Authors:** Zihao Guo, Ting Zeng, Yaoqin Lu, Shengzhi Sun, Xiao Liang, Jinjun Ran, Yushan Wu, Marc K. C. Chong, Kai Wang, Shi Zhao

**Affiliations:** 1 JC School of Public Health and Primary Care, Chinese University of Hong Kong, Hong Kong, China; 2 School of Public Health, Xinjiang Medical University, Urumqi, China; 3 State Key Laboratory of Pathogenesis, Prevention and Treatment of High Incidence Diseases in Central Asia, Department of Medical Engineering and Technology, Xinjiang Medical University, Urumqi, China; 4 Urumqi Center for Disease Control and Prevention, Urumqi, China; 5 Department of Epidemiology and Biostatistics, School of Public Health, Capital Medical University, Beijing, China; 6 Department of Rehabilitation Sciences, Hong Kong Polytechnic University, Hong Kong, China; 7 School of Public Health, Shanghai Jiao Tong University School of Medicine, Shanghai, China; 8 Centre for Health Systems and Policy Research, Chinese University of Hong Kong, Hong Kong, China; 9 Clinical Trials and Biostatistics Laboratory, CUHK Shenzhen Research Institute, Shenzhen, China; 10 School of Public Health, Tianjin Medical University, Tianjin, China; 11 Tianjin Key Laboratory of Environment, Nutrition and Public Health, Tianjin Medical University, Tianjin, China; 12 MoE Key Laboratory of Prevention and Control of Major Diseases in the Population, Tianjin Medical University, Tianjin, China

**Keywords:** Epidemiology, Viral infection

## Abstract

**Background:**

As SARS-CoV-2 Omicron variants circulating globally since 2022, assessing the transmission characteristics, and the protection of vaccines against emerging Omicron variants among children and adolescents are needed for guiding the control and vaccination policies.

**Methods:**

We conducted a retrospective cohort study for SARS-CoV-2 infections and close contacts aged <18 years from an outbreak seeded by Omicron BA.5 variants. The secondary attack rate (SAR) was calculated and the protective effects of two doses of inactivated vaccine (mainly Sinopharm /BBIBP-CorV) within a year versus one dose or two doses above a year after vaccination against the transmission and infection of Omicron BA.5 were estimated.

**Results:**

A total of 3442 all-age close contacts of 122 confirmed SARS-CoV-2 infections aged 0–17 years were included. The SAR was higher in the household setting and for individuals who received a one-dose inactivated vaccine or those who received a two-dose for more than one year, with estimates of 28.5% (95% credible interval [CrI]: 21.1, 37.7) and 55.3% (95% CrI: 24.4, 84.8), respectively. The second dose of inactivated vaccine conferred substantial protection against all infection and transmission of Omicron BA.5 variants within a year.

**Conclusions:**

Our findings support the rollout of the second dose of inactivated vaccine for children and adolescents during the Omciron BA.5 predominant epidemic phase. Given the continuous emergence of SARS-CoV-2 variants, monitoring the transmission risk and corresponding vaccine effectiveness against SARS-CoV-2 variants among children and adolescents is important to inform control strategy.

## Plain Language Summary

Children and adolescents have reported suffering less severe outcomes from the SARS-CoV-2 Omicron variant. However, the risk of transmission and vaccine effectiveness among this population group is not well studied. Here, we used contact tracing data that was collected during an Omicron BA.5 outbreak from Urumqi, China, before the exit of “zero-COVID” measures, to evaluate the spread of SARS-CoV-2 infection among those age under 18 years, and the effectiveness of inactivated vaccine regimens. Our findings indicate there is a high rate of transmission among children and adolescents in a household setting and receiving two doses of inactivated COVID-19 vaccination within a year was more effective than a single dose or two doses given more than a year apart. These findings highlight the importance of tracking transmission and vaccine effectiveness of novel SARS-CoV-2 variants in younger populations to inform control strategies.

## Abstract (web summary)

Guo, Zeng, Lu et al. assess SARS-CoV-2 Omicron BA.5 transmission and protection from inactivated vaccines among children and adolescents prior to the end of Zero-COVID measures in Urumqi, China. Two vaccine doses provide improved protection against Omicron infection and reduced transmission versus a single dose when given within the same year.

## Introduction

SARS-CoV-2 Omicron variants dominated the COVID-19 pandemic in 2022. They were found with increased transmissibility^1,2^, and resistance from both naturally acquired and vaccine-elicited antibodies^3,4^, which was considered to be one of the major challenges for disease control. The Omicron variants continuously evolved with epidemiologic and virological characteristics for adaptation in the human population. As of 8 January 2023, the Omicron BA.5 subvariants are the dominating strain amidst the pandemic, accounting for 85.9% of viral sequences of human SARS-CoV-2 submitted to the Global Initiative on Sharing Avian Influenza Data (GISAID) from 9 December 2022 to 9 January 2023^5^.

The increasing numbers of Omicron infections raised concerns, particularly for vulnerable populations including children and adolescents, who are often featured with higher contact rates (e.g., in school or household settings), and lower vaccine coverage than other age groups^6^. Although literature presented evidence suggesting that pediatric COVID-19 was rarely severe^7–9^, critical symptoms including the Multisystem Inflammatory Syndrome in Children (MIS-C)^8,10^ and long-COVID-19 may occur^11^. Besides, children and adolescents were found more susceptible to COVID-19 infection than adults, especially during an Omicron predominance phase^12,13^. Real-world observational studies demonstrated moderate effectiveness of mRNA COVID-19 vaccine (BNT162b2) against Omicron infection among children and adolescents^14,15^, and high vaccine effectiveness (VE) against hospitalization, death, and ICU admission^16–18^, though the protective effects waned quickly over time. To date, only a small number of studies have examined the VE of inactivated COVID-19 vaccines among children and adolescents^19–24^. Yet, none of these studies evaluated the VE against the Omicron subvariants (BA.5), which could provide additional insights into the protection of inactivated vaccines against the novel circulating SARS-CoV-2 variants.

From the standpoint of outbreak control, cutting off the transmission chain is one of the most effective measures apart from vaccination. Children and adolescents played an essential role in transmissions that occurred in household and school settings^25,26^. Despite early studies suggesting a lower transmission risk, as indicated by a lower secondary attack ratio (SAR) of children index cases than that of adult index cases for the ancestral SARS-CoV-2 strains^27^, children infected by the early Omicron variants were found more transmissible than adults^28^. Monitoring the transmission risks of children and adolescents infected by the emerging Omicron subvariants was urgently needed across different strata of risk factors and settings, given the rapid expansion of Omicron infections. Furthermore, the data regarding the VE against transmission among children and adolescents remains scarce^29,30^, which can shed light on the protection of vaccines against the risks of onward transmission seeded by children and adolescents.

From 1 August to 7 September 2022, Urumqi city, China experienced an outbreak seeded by the Omicron BA.5 variants. A series of stringent public health and social measures (PHSMs) have been implemented in response to the epidemics, including the city-wide lockdown, mass testing, contact tracing, and case isolation. Since August 3, 2021, China has recommended the inactivated vaccine rollout for children and adolescents aged 3–17 years, and Sinopharm (BBIBP-CorV) and Sinovac (CoronaVac) were the only two types of COVID-19 vaccine approved to be used in Urumqi city, where Sinopharm was the majority with >85% among vaccinated population (internal data from Urumqi CDC).

In this study, by leveraging detailed contact tracing data, we aim to assess the transmission characteristics of Omicron BA.5 among children and adolescents and the protective effectiveness of inactivated vaccines against the infection and transmission of the BA.5 variants. We estimate that the BA.5 variant has a relatively high risk of transmission among children and adolescents contacts in household while with considerable superspreading potential in non-household settings. We show that a second dose of inactivated vaccine within a year was associated with a substantial reduction in transmission risk in children and adolescents, as compared to a one-dose or dated second-dose vaccine. These findings underscore the need for monitoring the effectiveness of COVID-19 vaccines and the transmission potential among younger populations infected with emerging SARS-CoV-2 variants.

## Methods

### Study design and participants

This was a retrospective cohort analysis for SARS-CoV-2 infections and close contacts aged 0–17 years during an Omicron BA.5 outbreak in Urumqi, China. Children and adolescent close contacts and infected, and their close contacts were included in the screening. Owning to the zero-COVID policy (implemented before November 2022) in mainland China, no large-scale COVID-19 outbreak occurred in Urumqi before August 2022, which means that the 3.8 million population was largely infection-naïve, and thereby the likelihood of re-infection was negligible. The COVID-19 vaccines received by almost all vaccinees in mainland China were Sinopharm and Sinovac vaccines, which were recommended and administered under the supervision of Chinese authorities for those aged over 3 years.

De-identified individual-level line-list surveillance data was obtained from the Xinjiang Uygur Autonomous Region Health Committee from August 1 to September 7, 2022, covering an entire Omicron BA.5 outbreak. Demographic and epidemiological information was collected including the sex, age, contact tracing history, date of exposure, reverse transcription polymerase chain reaction (RT-PCR) test-positive date of each test-positive contact (i.e., COVID-19 case), and last test-negative date of each test-negative contact, contact setting (i.e., household, and non-household settings), and doses and dates of vaccination before test-positive or exposure. The definitions of COVID-19 cases and close contacts were detailed in Supplementary Note 1. Based on the individual’s reported exposure and contact tracing history, the number of secondary cases per index case was extracted for analysis of the transmission risk. During the study period, no study participant received a third dose of vaccine. As the majority (91%, 362 out of 399 in total) of the unvaccinated children in our dataset were aged ≤ 3 years, to which age group COVID-19 vaccine was not recommended in mainland China^31^, these children would not be representative samples of unvaccinated children and adolescents. We thus removed unvaccinated children from analyses and restricted our attention to the VE of two-dose against one-dose vaccines. In view of the small sample size for close contacts who received only one-dose vaccine, we considered individuals who received two-dose vaccine over 365 days as a complement of the reference group, assuming the vaccine-elicited immunity remained after 1 year was at a relatively low level^32,33^.

The follow-up for each subject started from the initial date of the local outbreak on August 1, 2022, till the test-positive date for test-positive individuals, or till the loss of follow-up (e.g., right-truncated) at the last test-negative date for test-negative individuals. The date of exposure for close contacts was defined as the time point of the first contact to index cases within the infectious period^29^, which was the time window starting from 4 days before and ended to 8 days after the test-positive date. Individuals with unknown exposure dates, and individuals who received the last vaccine dose within 14 days before test-positive for test-positive contacts or before exposure for test-negative contacts were excluded from the analysis of VE^34^.

### Statistical analyses

To assess the transmission risks, the secondary attack ratio (SAR) was calculated by fitting a beta-binomial model to close contact data to address the possible individual heterogeneity^35^. In addition, following classic epidemiological theory ^36^, we jointly estimated the reproduction number (*R*), defined as the average number of secondary cases seeded by a single index case, and a dispersion parameter (*k*), measuring the heterogeneity of the individual reproduction number by using the negative binomial distribution, which was a widely adopted method during the COVID-19 epidemics^37–39^. Stratified estimations for the *R*, *k*, and SAR were performed based on the characteristics of the index case, including the sex, age (preschool children: 0–5 years, primary school children: 6–12 years, and adolescent: 13–17 years), vaccination status, and symptom status (Supplementary Note 2) and the contact setting where the close contacts occurred and epidemic phase (before and after city lockdown measurement). The *R*, *k*, and SAR were estimated in a Bayesian statistical inference framework by using the Metropolis-Hasting Markov chain Monte Carlo (MCMC) method. The 95% credible intervals (CrI) of the parameters’ estimates were generated by finding the 2.5-th and 97.5-th percentiles of converged posterior distributions. The details of the statistical model were described in Supplementary Note 3.

For the analysis of effectiveness of inactivated vaccines against Omicron BA.5 variant, we focused on comparing the VE of two-dose inactivated vaccine with different lag (from the time point of receiving the last dose to the RT-PCR testing date; two-dose vaccinees with a lag of 15–180, and 181–365 days) to our predefined reference group (all one-dose vaccinees, and two-dose vaccinees with a lag longer than 365 days). Descriptive statistics for the vaccinated close contacts stratified by the vaccination status were drawn to characterize the cohort for studying the VE. The odds ratios (ORs) of vaccine status against all infection, symptomatic infection only, and transmission were estimated by using the Bayesian logistic regression models. The VE was defined as (1−OR) × 100% when OR < 1, or as − (1−1/OR) × 100% when OR > 1^40,41^. We included the following covariables in the multivariate regression models to control for confounding effects: age, sex, contact setting, vaccine dosage of index case or contacts, and the calendar date of contact (exposure). Both crude and adjusted VE were presented, and the VE estimates were summarized as median and 95% CrI of the estimated posterior distributions.

We performed subgroup analyses according to age groups (children with age 0–12 years, and adolescents with age 13–17 years), contact setting, and vaccination status of the index case (0–2 doses of vaccine, and 3 doses of vaccine). All statistical analyses were performed in **R** statistical software, version 4.1.3 (R Foundation for Statistical Computing).

### Ethical approval

The collection of specimens, epidemiological and clinical data for SARS-CoV-2 infected individuals and their close contacts is part of a continuing public health investigation of COVID-19 outbreaks, ruled in the Protocol on the Prevention and Control of COVID-19 by the National Health Commission of the People’s Republic of China, which was exempt from ethical approval (i.e., institutional review board assessment). This study was approved by the institutional ethics committee of Xinjiang Medical University (IRB No.: XJYKDXR20221001001). Because this study was a retrospective analysis using secondary data without personal identity or human samples, the requirement for obtaining informed consent was waived.

### Reporting summary

Further information on research design is available in the Nature Portfolio Reporting Summary linked to this article.

## Results

Between August 1 to September 7, 2022, a total of 1139 RT-PCR-confirmed cases (including 23 imported cases, 83 symptomatic cases, and 1033 asymptomatic cases) were reported in Urumqi, China. We identified a total of 51786 close contacts from 769 index cases during the study period, among which 3442 close contacts (80 test-positive contacts, 3362 test-negative contacts; mean [SD] age of 35 [19] years; 1666 males [48.4%]) of the 122 index cases with ages 0–17 years (mean [SD] age of 10 [4] years; 71 males [78.2%]) were analyzed. In addition, 104 cases with ages 0–17 years who had zero close contact, likely due to the rapid case identification and isolation (thus a total of 226 confirmed pediatric cases).

### Characteristics of transmission risks

We estimated the *R* and *k* of the 80 secondary cases from the 226 primary cases (including 122 index cases that have at least one close contact and 104 cases that have zero close contact) to be 0.37 (95% CrI: 0.26, 0.52) and 0.24 (95%CrI: 0.14, 0.43), with an expected 12.5% (95% CrI: 9.2, 16.4) of index cases generating 80% of the transmission events. After fitting the beta-binomial distribution to the number of positive contacts, the mean SAR for overall index cases was estimated at 13.7% (95% CrI: 8.9, 20.2), with the 95-th percentile of the distribution estimated at 79.1% (95% CrI: 51.1, 97.3), see Table 1. The *R* estimates remained similar among different age groups, whereas the transmission from preschool children appeared to be the most heterogeneous, with only 6.8% (95% CrI: 2.6, 13.7) cases responsible for 80% of all transmission events. The adolescent case group had the lowest mean SAR and a relatively larger coefficient of variation (CV) estimates among all age groups (Table 1). In addition, the SAR and *R* estimates were higher in the household setting and during the post-lockdown phase, though the *k* estimates were relatively higher (lower transmission heterogeneity). Compared to the index cases with one-dose vaccine or two-dose vaccine for more than a year, those who received a second dose of vaccine within a year had substantially lower *R* and mean SAR estimates, though the transmission heterogeneity was at similar levels in terms of *k*. The mean SAR and *R* estimates appeared close between symptomatic and asymptomatic cases. The parameter estimates for the beta-binomial model are shown in Supplementary Table 1.

**Table 1:** The estimated characteristics of transmission risks of 122 index children and adolescent cases between 1 August to 7 September 2022 in Urumqi, China

Characteristics	Sample size (column %)			Secondary attack ratio (95% CrI)			Transmission and superspreading potentials (95% CrI)		
	Index case (%)^a^	Close contact							
		Test-positive (%)	Total (%)	Mean	CV	95-th percentile	Reproduction number	Dispersion parameter	Prop80%^b^
Total	122 (100.0)	80 (100.0)	3442 (100.0)	13.7% (8.9, 20.2)	1.8 (1.5, 2.2)	79.1% (51.1, 97.3)	0.37 (0.26, 0.52)	0.24 (0.14, 0.43)	12.5% (9.2, 16.4)
Sex of index case									
Male	71 (58.2)	50 (62.5)	2331 (67.7)	11.1% (6.0, 18.8)	1.9 (1.5, 2.4)	65.7% (32.9, 95.6)	0.40 (0.25, 0.68)	0.18 (0.19, 0.36)	10.9% (7.0, 15.7)
Female	51 (41.8)	30 (37.5)	1111 (32.3)	14.8% (6.6, 26.3)	1.6 (1.3, 2.1)	78.3% (28.3, 99.3)	0.35 (0.22, 0.59)	0.40 (0.16, 1.42)	14.9% (9.5, 21.6)
Age of index case									
Preschool children: 0–5 yr	15 (12.3)	14 (17.5)	661 (28.4)	17.3% (4.9, 43.2)	1.5 (1.0, 2.5)	88.4% (23.4, 100)	0.32 (0.11, 1.18)	0.09 (0.03, 0.30)	6.8% (2.6, 13.7)
Primary school children: 6–12 yr	67 (54.9)	41 (51.2)	1685 (24.6)	13.9% (8.2, 22.9)	1.7 (1.4, 2.1)	72.5% (42.8, 97.7)	0.38 (0.25, 0.54)	0.90 (0.31, 3.62)	19.1% (13.4, 24.8)
Adolescent: 13–17 yr	40 (32.8)	25 (31.3)	1096 (47.0)	8.7% (3.7, 18.2)	2.2 (1.6, 3.1)	58.9% (23.8, 98.9)	0.38 (0.18, 1.01)	0.12 (0.04, 0.31)	8.1% (4.1, 14.1)
Epidemic phase									
Before lockdown	34 (27.9)	8 (10.0)	1004 (29.2)	1.4% (0.6, 3.0)	2.0 (1.4, 3.3)	6.9% (3.3, 13.6)	0.16 (0.06, 0.44)	0.31 (0.05, 10.84)	8.1% (3.1, 15.7)
After lockdown	88 (72.1)	72 (90.0)	2438 (70.8)	17.0% (10.8, 25.1)	1.6 (1.3, 1.9)	82.2% (54.6, 98.4)	0.43 (0.29, 0.64)	0.25 (0.14, 0.46)	13.3% (9.5, 17.8)
Contact setting									
Household	90^c^	54 (67.5)	234 (6.8)	28.5% (21.1, 37.7)	1.0 (0.7, 1.3)	85.9% (60.9, 98.9)	0.25 (0.17, 0.35)	0.41 (0.20, 1.15)	12.5% (9.1, 16.5)
Non-household	100^c^	26 (32.5)	3208 (93.2)	2.6% (1.1, 6.5)	3.4 (2.5, 4.9)	15.9% (5.4, 58.7)	0.14 (0.06, 0.40)	0.04 (0.02, 0.08)	2.9% (1.5, 5.0)
Vaccine status of index case, stratified by lag from the last dose to SARS-CoV-2 infection									
2 doses with lag 15–365 d	96^d^	47	2659	9.6% (5.1, 16.3)	1.9 (1.6, 2.3)	55.8% (26.1, 89.1)	0.50 (0.35, 0.70)	1.53 (0.45, 6.64)	24.8% (17.8, 31.3)
1 dose & 2 doses with lag 365+ d	8^d^	21	96	55.3% (24.4, 84.8)	0.7 (0.3, 1.4)	99.7% (74.8, 100)	3.46 (1.30, 11.46)	0.63 (0.15, 3.03)	31.7% (12.7, 49.9)
Status of symptoms of index cases									
Asymptomatic or mildly symptomatic	109 (89.3)	70 (87.5)	2952 (85.8)	12.3% (7.4, 19.1)	1.9 (1.6, 2.3)	73.8% (43.2, 96.9)	0.41 (0.20, 0.73)	8.22 (1.59, 16.64)	24.5 (13.7, 34.8)
Moderately symptomatic or severe	13 (10.7)	10 (12.5)	490 (14.2)	15.9% (6.3, 37.1)	1.2 (0.8, 1.9)	60.8% (25.5, 99.3)	0.37 (0.25, 0.56)	0.19 (0.11, 0.34)	11.0 (7.8, 14.9)

^a^The cases who had close contacts. Another 104 cases who had zero close contact were only included in the estimation of the reproduction number.

^b^The expected proportion of index cases generating 80% of all transmission events.

^c^Including the number of index cases who had secondary cases in both household and non-household settings, and thus 90 + 100 > 122.

^d^Index cases with missing exposure date were excluded.

### VE against Omicron infection and transmission

**Fig. 1:**
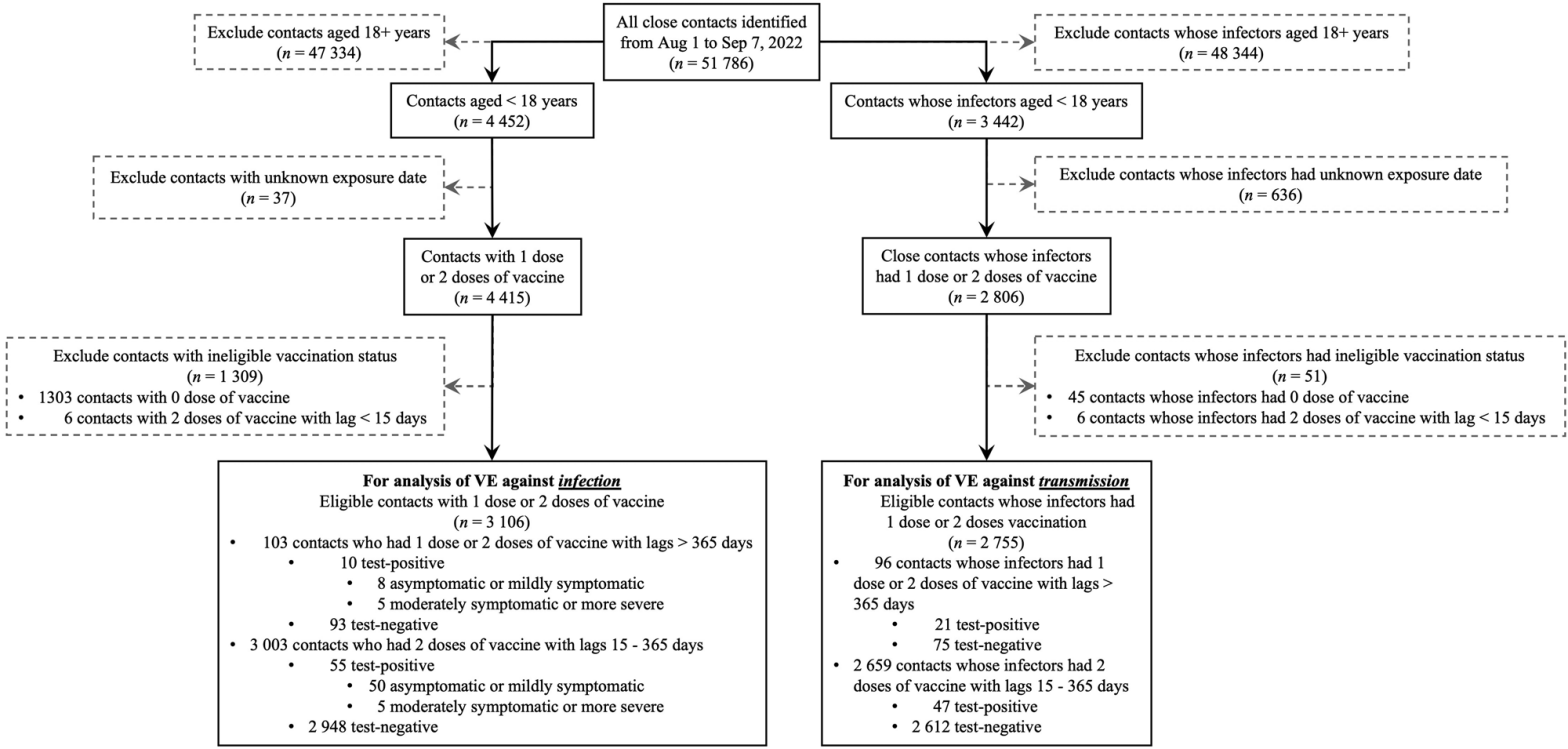
Flowchart of sample selection of the study. This diagram illustrates the process for identifying eligible study participants for assessing the protective effects of vaccine against infection (the left arm) and against transmission (the right arm).

For the analysis of VE against infection we included 3106 eligible close contacts (mean [SD] age of 11 [4] years; 1639 males [52.8%]), of which 103 received one-dose vaccine or received a two-dose vaccine but with a lag of more than 365 days before the RT-PCR testing date, and 3003 had a two-dose vaccine with a lag of 15–365 days. Figure 1 showed the selection process of the study cohort, and the characteristics of the included close contacts were presented in Table 2. Only age was significantly different between one-dose (mean age of 8 years) and two-dose vaccinees (mean age of 11 years) groups. The proportion of preschool children was higher whereas the fractions of primary-school children and adolescents were lower for the one-dose group than for the two-dose group (Table 2). Using close contacts who had one dose of vaccine or those who had two doses of vaccine for more than 365 days as the reference group in the multivariate regression model, a second dose of inactivated vaccine provided effectiveness of 92.4% (95% CrI: 46.8, 98.9) against Omicron infection, regardless of symptom, within 180 days. The VE against all infection attenuated to 75.4% (95% CrI: 43.4, 89.3) 181–365 days after receiving the second dose, see Supplementary Data 1. In the subgroup analyses, the VE of a two-dose vaccine was estimated at 85.5% (95% CrI: 49.1, 95.9) for children aged 0–12 years; VE among close contacts whose index cases did not receive a booster dose was 84.1% (95% CrI: 57.3, 94.1); VE among close contacts exposed in the household setting was estimated at 78.0% (95% CrI: 20.9, 93.9), which was higher than in non-household setting. A considerably higher VE against the moderately symptomatic or more severe Omicron infection was observed for children aged 3–12 years receiving the second dose of vaccine within 365 days (90.7%; 95% CrI: 84.2, 94.5). Among 3442 close contacts of 122 index cases identified during the study period, 2755 eligible contacts (mean [SD] age of 36 [19] years; 1372 males [49.8%]) of 104 cases aged 0–17 years (mean [SD] age of 11 [4] years; 63 males [60.6%]) were included for the analysis of VE against transmission. The VE for the second dose of vaccine within 365 days was 95.6% (95% CrI: 90.4, 98.0) against the transmission of Omicron BA.5 seeded by index cases aged below 18 years. In subgroup analysis, the uncertainty interval of VE crossed zero (i.e., the vaccine’s positive effect is not certain) for adolescent index cases (77.3%; 95% CrI: −47.9, 97.3) but not for children index cases (98.1%; 95% CrI: 90.8, 99.6), and for index cases whose close contact occurred in non-household settings (91.6%; 95% CrI: 72.1, 97.5; Supplementary Data 1).

**Table 2:** Baseline characteristics of identified children and adolescent close contacts who have received at least one dose of COVID-19 vaccines

Characteristics of contacts	Sample size (column %)					*p*-value^b^ for 1 vs. 2 doses
	1 dose	2 doses, by lag from the 2nd dose to exposure				
		15–180 d	181–365 d	365+ d	Total^a^	
Total (%)	37 (100)	179 (100)	2824 (100)	66 (100)	3069 (100)	NA
Sex						
Male (%)	22 (59)	96 (54)	1488 (53)	33 (50)	1617 (53)	0.510
Female (%)	15 (41)	83 (46)	1336 (47)	33 (50)	1452 (47)	
Age group						
Preschool children: 0 - 5 yr (%)	22 (59)	91 (51)	182 (6)	2 (3)	275 (9)	<0.001
Primary school children: 6 - 12 yr (%)	4 (11)	74 (41)	1546 (55)	2 (3)	1622 (53)	
Adolescent: 13 - 17 yr (%)	11 (30)	14 (8)	1096 (39)	62 (94)	1172 (38)	
Mean age, yr (SD)	8 (5.3)	6 (3.2)	11 (3.7)	15 (2.6)	11 (3.9)	<0.001
Epidemiological week of 2022						
wk 31: Jul 31–Aug 6 (%)	7 (19)	47 (26)	647 (23)	0 (0)	694 (23)	0.995
wk 32: Aug 7–Aug 13 (%)	14 (38)	65 (36)	1151 (41)	0 (0)	1216 (40)	
wk 33: Aug 14–Aug 20 (%)	6 (16)	24 (14)	443 (16)	1 (2)	468 (15)	
wk 34: Aug 21–Aug 27 (%)	6 (16)	29 (16)	398 (14)	18 (27)	445 (14)	
wk 35: Aug 28–Sep 3 (%)	3 (8)	13 (7)	146 (5)	35 (53)	194 (6)	
wk 36: Sep 4–Sep 10 (%)	1 (3)	1 (1)	39 (1)	12 (18)	52 (2)	
Contact setting						
Household (%)	3 (8)	9 (5)	210 (7)	10 (15)	229 (7)	0.997
Community (%)	1 (3)	6 (3)	111 (4)	1 (2)	118 (4)	
Work place (%)	0 (0)	0 (0)	1 (1)	0 (0)	1 (1)	
Unknown settings (%)	33 (89)	164 (92)	2502 (88)	55 (83)	2721 (88)	
Age group of source case						
Minor: <18 yr (%)	8 (22)	36 (20)	507 (18)	16 (24)	559 (18)	0.310
Young adult: 18–39 yr (%)	18 (49)	65 (36)	1120 (40)	30 (46)	1215 (40)	
Middle-age adult: 40–64 yr (%)	11 (29)	69 (39)	1041 (37)	16 (24)	1126 (37)	
Old-age adult: 65+ yr (%)	0 (0)	9 (5)	156 (5)	4 (6)	169 (5)	
Mean age of source case, yr (SD)	31 (15.7)	35 (18.0)	35 (17.4)	35 (18.3)	35 (17.3)	0.190
Vaccine status of source case						
0–1 dose (%)	4 (11)	13 (7)	214 (8)	7 (11)	234 (8)	0.710
2 doses (%)	8 (22)	58 (33)	724 (26)	21 (32)	803 (26)	
3 doses (%)	25 (67)	108 (60)	1886 (66)	38 (57)	2032 (66)	

^a^The total sample size of two-dose vaccinees here included those vaccinated within 14 days before the test-positive date or the latest test date with a test-negative outcome.

^b^The comparison of mean and frequency between the 1-dose group, and the 2-dose group (total) was done by using the Chi-squared test or Fisher’s exact test with a significance level of *p*-value =  0.05.

## Discussion

To effectively respond to the fast dissemination of SARS-CoV-2 infections triggered by the novel Omicron variants, it is crucial to evaluate the transmission risk and vaccine effectiveness among children and adolescents, who normally have lower vaccination coverage and higher contact rates compared to adult populations. Here, we utilized detailed contact tracing and epidemiological data to characterize the transmission risk and assess the protective effectiveness of inactivated COVID-19 vaccines against infection and transmission in younger populations infected with the Omicron BA.5 infection.

Our analysis of transmission risk suggested an overall modest transmissibility and probability of infection per contact for all 122 identified children and adolescent cases with Omicron BA.5 infection. The transmissibility remained similar for cases with different ages, as indicated by the similar *R* estimates. However, the transmission appeared to be more heterogeneous (with a smaller *k* value) for children aged 0–5 and adolescents aged 13–17 than for children aged 6–12 years. A lower *k* value suggested an over-dispersed secondary case distribution, which was characterized by an excessive zero-class, representing that a substantial fraction of index cases led to zero secondary cases, or a long tail, favoring the risks of superspreading^42^. Although the *R* estimate remained low and similar in both household and non-household settings, the mean SAR estimate was considerably high in the household setting. This could be attributed to the rapidly imposed lockdown measures that restricted the majority of transmission events occurring within households, as suggested by the higher SAR observed in the post-lockdown phase than in the pre-lockdown phase (Table 1). Early studies on the risk of transmission from children in different settings showed that children may play a lesser role in COVID-19 transmission than adults^26,27,43^, possibly because children had lower within-host viral load^44^ and are less susceptible to the SARS-CoV-2 infection than adults^27^. Considering children were frequently reported as asymptomatic cases^45,46^, which may be responsible for widespread infection even when PHSMs were in place, it is essential to enhance household surveillance especially when the PHSMs were relaxed, to avoid a resurgence of the epidemics^45^. The lower value of *k* estimates in non-household settings was corroborated by a previous study analyzing the transmission chains during an Omicron outbreak in South Korea^38^, where the transmission occurred in community settings had a lower *k* value than the household setting. A sufficiently smaller *k* value indicated a higher potential of superspreading events, which was less sensitive to the PHSMs^37,39^. The role of children and adolescent COVID-19 cases in generating superspreading events is unclear and the vaccine effectiveness in preventing superspreading events needs further investigations.

Variable estimates of VE against Omicron infection among the pediatric population for the inactivated vaccine have been reported in different countries and regions. In Argentina, Castelli et al. reported a point VE estimate of 16% for the primary series of Sinopharm vaccine among 3–11 years old^23^. Data from Chile^21^ and Brazil^19^ suggested the VE of two-dose CoronaVac were 38.2% and 39.8% for 3–5 years old and 6–11 years old, respectively. The disagreements between the VE estimates from these studies and ours may largely attributed to the context of the study setting. China has been implementing a strict zero COVID-19 strategy during the course of the Omicron epidemic when control measures including city-wide lockdown, social distancing, and closure of common public places (e.g., workplaces, schools, shopping marts, restaurants, and entertainment settings) were conducted. Thus, the number of exposure or contact was generally lower in the population, which gave rise to a lower risk of infection and transmission. A study has shown that the protection conferred by the COVID-19 vaccine is dependent on the rate of exposure, with a higher level of exposure diminishing the effectiveness of vaccine against the Omicron infection^47^. Our VE estimates were also higher than that from a Hong Kong study, which indicated that the effectiveness of a two-dose CoronaVac vaccine was 55% against infection among 12–18 years old^20^. Since the beginning of the Omicron epidemic, Hong Kong has implemented a Vaccine Pass policy for individuals over 12 years old, whereby at least one dose of vaccine was required for entering public facilities. As such, vaccinated adolescents likely had a higher risk of infection because of being involved in various exposure settings as compared to an unvaccinated one, which may drive the VE downward. Moreover, previous data demonstrated that the Sinopharm vaccine had a higher relative effectiveness against SARS-CoV-2 infection than the Sinovac-CoronaVac vaccine^48^, which may also contribute to explaining the difference in VE of the present study and that of the Brazilian and Chilean studies, given that the majority of the participants (>85%) received the Sinopharm vaccine. Additionally, a comparative study among adolescents aged 12–17 years old showed the effectiveness of a two-dose BNT162b2 vaccine against infection was much higher in Scotland (80.7%) than in Brazil (64.7%), and the VE waned at a much lower rate in Scotland compared to Brazil^49^. Therefore, the heterogeneity of the underlying population could also play an important role in determining the VE estimates. Our analysis for VE against the Omicron BA.5 infection showed that among children and adolescents, the risk of infection by the BA.5 variants for individuals who received a two-dose inactivated vaccine reduced by 92.4% within six months (75.4% for those who have received a two-dose inactivated vaccine for 181–365 days) (Supplementary Data 1), as compared to those who only received a one-dose inactivated vaccine and those who received the two-dose regimen for more than a year. Considering a one-dose vaccine only provided a weak immune response and protection against SARS-CoV-2 infection, and the immunity level would wane to a considerably lower level a year after receiving the second dose of the inactivated vaccine^19,32,33,50^, our reference group might represent the group of children and adolescents who have a relatively lower internal immunity induced by the vaccine. Furthermore, the estimated risk reduction not only reflected the additional protection conferred by a second dose of vaccine compared to a one-dose vaccine but also a waned VE. Given that the effectiveness of the inactivated vaccine against the Omicron variant tends to wane rapidly within months after the inoculation among children and adolescents^23^, along with the compromised immune response induced by the vaccine^51^, a booster dose of COVID-19 vaccine should be considered for the pediatric population in China. To our knowledge, only a few studies evaluated the VE of mRNA vaccines against the transmission during the period when SARS-CoV-2 Alpha or Delta variants dominated^29,30,52^, and the investigation of VE of inactivated vaccines against transmission of Omicron subvariants was generally lacking^53^. Notably, our findings suggested an effective protection against BA.5 transmission within a year conferred by a second dose of inactivated vaccine in children and adolescents, consistent with another finding of lower values of *R* and SAR estimates for children and adolescents index cases with two-dose vaccine within a year. Furthermore, it appeared that the level of VE against transmission sustained within a year post-vaccination, partly consistent with the finding from large-scale cohort studies suggesting that the protection provided by the vaccine against individual infectiousness was less affected by time^54^.

Our study had several limitations. First, our estimations of the transmission risk and VE relied heavily on contact tracing data. Thus, any recall bias from the traced cases during the contact tracing process, and the under-ascertainment of case issues would deviate our estimates. Nonetheless, since lockdown and door-to-door mass testing have been rapidly conducted since the very beginning of the local outbreak, the proportion of under-ascertainment cases was assumed to be low. Second, selection bias may affect the VE estimates if there were systematic differences between the two-dose vaccine and reference groups. We partially accounted for this issue by adjusting the estimates with several observable confounders. Nevertheless, limited access to the data restricted us from testing whether children and adolescent or their caregivers in these groups contrast in some unobservable characteristics such as risk perception to infection and compliance to COVID-19 prevention behaviors. The sample size for the analyses of VE was small and may not be representative of the general pediatric population in China and thereby our VE estimates should be interpreted with caution. Our study sample contained all pediatric cases and close contacts identified during the whole course of an Omicron outbreak and we believed that our finding would be especially helpful for the local government and other settings with similar public health capacity. In addition, as we lack data regarding the vaccine brand (Sinopharm or Sinovac) each participant received, we considered two inactivated vaccine brands together in the analysis. Our VE estimates were mostly driven by the Sinopharm vaccine, given that majority of the vaccinees received the Sinopharm vaccine during the study period. Third, since no children and adolescents in our study cohort received a third dose of vaccine, we cannot assess the effectiveness of a booster dose of inactivated vaccine. Furthermore, since we did not have unbiased unvaccinated children and adolescents, we cannot estimate the absolute effectiveness of a two-dose inactivated vaccine. Finally, as the majority of children and adolescent cases included in our study were asymptomatic (89%, 109/122), the estimated VE may be more generalizable to asymptomatic cases. Likewise, as the most of subjects in the 0–12 age group were over 3 years old, our findings for this age group should be interpreted towards those aged 4–12 years.

## Conclusions

Our findings demonstrated a moderate transmission risk of children and adolescent cases with the Omicron BA.5 infection, with a higher risk in household settings in the context of stringent PHSMs. Two-dose inactivated vaccine may potentially reduce the risk of transmission. The protective effect of a two-dose inactivated vaccine against all infection and transmission of Omicron BA.5 was high among children and adolescents within a year compared to a one-dose or a two-dose vaccine after a year. These results provide additional evidence concerning the inactivated vaccine to prevent the Omicron BA.5 infection and transmission. Considering the persistently emerging Omicron variants, monitoring the effectiveness of COVID-19 vaccines is important to inform vaccine rollout among children and adolescents.

### Supplementary information

Supplementary information

Description of Additional Supplementary Files

Supplementary Data 1

Reporting Summary

Supplementary Data 2

## Data Availability

The anonymized data for generating the tables is available in Supplementary Data 2.

## Code availability

Statistical analyses were performed in R (Version 4.1.3). The code is available at 10.5281/zenodo.10518983^55^.
