# Supplementary material for: Transmission risks of Omicron BA.5 following inactivated COVID-19 vaccines among children and adolescents in China
Source: Commun Med (Lond). 2024 May 18;4:92. doi: 10.1038/s43856-024-00521-y (PMC11102477; doi:10.1038/s43856-024-00521-y)
Supplement: Supplementary file 1 — Supplementary information [file 43856_2024_521_MOESM1_ESM.docx]

**Supplementary Information**

Table of Contents

[**Supplementary Note 1. COVID-19 cases and close contacts during the Omicron BA.5 outbreak** 2](#_Toc164784587)

[**Supplementary Note 2. Outcome variables** 3](#_Toc164784588)

[**Supplementary Note 3. Statistical models** 4](#_Toc164784589)

[**Supplementary Table 1. Estimated parameter values of the beta-binomial model.** 6](#_Toc164784590)

[**References** 7](#_Toc164784591)

## **Supplementary Note 1. COVID-19 cases and close contacts during the Omicron BA.5 outbreak**

City-wide lockdown was imposed within 4 days after the detection of the first local case on August 7, 2022. Since then, door-to-door real-time reverse transcription polymerase chain reaction (RT-PCR) testing and contact tracing were conducted to identify possible cases. Individuals undergone the RT-PCR with a cycle threshold (Ct) value lower than 40 were considered as test-positive case (SARS-CoV-2 infection). We included all close contacts and cases aged below 18 years with dates of contact, or dates of test-positive, respectively, ranging from August 1 to September 7, 2022, which covered the period of this city-wide COVID-19 outbreak.

Close contacts were defined as someone who had prolonged interaction with a confirmed case, i.e., more than one hour if both persons wear masks or ten minutes without masks usage. RT-PCR test was performed for each close contact immediately after tracing the index case based on contact history. All COVID-19 cases were confirmed by RT-PCR tests on a nasopharyngeal swab. The tracing and detecting of COVID-19 cases and close contacts were performed by healthcare professional or public health practitioners as a mandatory procedure of “zero-COVID” policy and under the supervision of local Center for Disease Control and Prevention (CDC).

## **Supplementary Note 2. Outcome variables**

The primary outcome for assessing the VE against Omicron infection was RT-PCR confirmed Omicron infection, regardless of symptom status. The secondary outcome here was Omicron infection with moderately symptomatic or severe outcomes. The moderately symptomatic or severe outcomes were defined as RT-PCR test positive cases with any respiratory symptoms that continuously presented for over 7 days or with radiographic presentation of pneumonia. Symptomatic cases with other milder symptoms were classified as mildly symptomatic cases. Asymptomatic cases were test-positive cases without any clinically evident symptoms at the time of testing.

The primary outcomes for assessing the VE against Omicron transmission was the number of secondary cases out of total number of identified close contacts for each index cases, which was used to calculate the secondary attack ratio (SAR).

## **Supplementary Note 3. Statistical models**

We characterized the transmission risks and heterogeneities in contact and transmission using the following statistical approaches. Considering the stochastic effect of transmission events, we assumed the number of secondary cases generated by the index cases followed a negative binomial distribution, which is parametrized by a reproduction number (*R*), defined as the average number of secondary cases seeded by a single index case, and a dispersion parameter (*k*), measuring the heterogeneity of the individual reproduction number (1). A smaller *k* value suggested a higher transmission heterogeneity. The probability mass function of negative binomial distribution is:

$$Pr\left( Z=z;R,k \right)=\frac{\Gamma\left( k+z \right)}{\Gamma\left( k \right) \Gamma\left( z+1 \right)}\left( \frac{R}{R+k} \right)^{z}\left( \frac{k}{R+k} \right)^{k}$$

Here, Γ(.) denotes the gamma function, *z* is the number of secondary cases generated by an index case. Armed with the *R* and *k* estimates, we calculated the expected proportion of index case responsible for generating 80% of total transmission (2), which is an empirical measurement of superspreading potential (3), given by:

$$1-P=\int_{0}^{Z} Pr\left( Z=z;R,k \right)dz$$

where *Z* satisfies:

$$1-80\%= \frac{1}{R}\int_{0}^{Z} \left\lfloor z \right\rfloor Pr\left( Z=z;R,k \right)dz$$

Here, $\left\lfloor z \right\rfloor$ denoted the floor function.

Additionally, we estimated the probability of infection per contact, that is, the SAR. We assumed the number of test positive contact $k_{i}$ out of total number of close contacts $n_{i}$ of the index cases $i$ followed a binomial distribution, conditioning on the SAR, i.e., $P_{i}$. To take account of the individual variations in SAR, we assumed the random variable $P_{i}$ followed a Beta distribution that parametrized by two shape parameters $\alpha$ and $\beta$. The mean of was $\frac{\alpha}{\alpha+\beta}$. This would thus yield a beta-binomial distribution for $k_{i}$, which is given by (4):


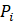

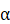

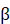

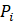

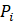

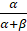

$$\Pr\left( K=k_{i};N=n_{i},\alpha,\beta\right)=\frac{\binom{n_{i}}{k_{i}}\mathrm{Be}(k_{i}+\alpha,n_{i}-k_{i}+\beta)}{\mathrm{Be}(\alpha,\beta)}$$

Here, $\mathrm{Be}\left( \alpha,\beta\right)=\int_{0}^{1} x^{\alpha-1}{(1-x)}^{\beta-1}dx$, which is the beta function. $K$ denotes the number of secondary cases and $N$ denotes the number of close contacts of an index case. The mean, coefficient of variation (CV), and 95-th percentiles were calculated for the fitted beta-binomial distributions. The *R*, *k*, and SAR were estimated by using the Metropolis-Hasting Markov chain Monte Carlo (MCMC) method, which was a widely adopted Bayesian statistical inference framework. Non-informative priors were used, and the marginal posterior distributions were obtained from 100000 MCMC iterations, among which the first 40000 samples were discarded as burn-in period. The convergence of each MCMC chain was checked by using the trace plot and Gelman-Rubin-Brooks convergence diagnostic (5).

## **Supplementary Table 1. Estimated parameter values of the beta-binomial model.**

| Characteristics | Parameters of the Beta-binomial model (95% CrI) | |
| --- | --- | --- |
|  |  |  |
|  | Shape parameter, α | Shape parameter, β |
| **Total** | 0.12 (0.08, 0.19) | 0.76 (0.38, 1.60) |
| **Sex of index case** | | |
| male | 0.13 (0.07, 0.23) | 1.0 (0.41, 2.81) |
| female | 0.15 (0.08, 0.36) | 0.85 (0.32, 5.03) |
| **Age of index case** | | |
| pre-school children: 3 - 5 yr | 0.13 (0.03, 0.48) | 0.62 (0.08, 5.53) |
| primary school children: 6 - 12 yr | 0.17 (0.09, 0.27) | 1.01 (0.40, 2.46) |
| adolescent: 13 - 17 yr | 0.09 (0.04, 0.21) | 0.95 (0.26, 2.95) |
| **Epidemic phase** | | |
| before lockdown | 0.23 (0.09, 0.48) | 17.7 (6.90, 22.74) |
| after lockdown | 0.17 (0.10, 0.28) | 0.83 (0.39, 1.76) |
| **Contact setting** | | |
| household | 0.44 (0.20, 1.16) | 1.10 (0.45, 3.22) |
| non-household | 0.05 (0.02, 0.13) | 1.79 (0.40, 8.92) |
| **Vaccine status of index case, stratified by lag from the last dose to SARS-CoV-2 infection** | | |
| 2 doses with lag 15 - 365 d | 0.24 (0.03, 1.27) | 0.19 (0.02, 2.14) |
| 1 dose & 2 doses with lag 365+ d | 0.14 (0.08, 0.27) | 1.31 (0.54, 4.02) |
| **Status of symptoms of index cases** | | |
| asymptomatic or mildly symptomatic | 0.12 (0.07, 0.19) | 0.83 (0.37, 1.89) |
| moderately symptomatic or severe | 0.36 (0.13, 0.90) | 1.91 (0.38, 7.25) |

## **References**

1 Lloyd-Smith JO, Schreiber SJ, Kopp PE, Getz WM. Superspreading and the effect of individual variation on disease emergence. Nature. 2005;438:355–9.

2 Endo A, Abbott S, Kucharski AJ, Funk S, Centre for the Mathematical Modelling of Infectious Diseases COVID-19 Working Group. Estimating the overdispersion in COVID-19 transmission using outbreak sizes outside China. Wellcome Open Res. 2020;5:67.

3 Guo Z, Zhao S, Lee SS, Mok CKP, Wong NS, Wang J, et al. Superspreading potential of COVID-19 outbreak seeded by Omicron variants of SARS-CoV-2 in Hong Kong. J Travel Med. 2022;29.

4 Prentice RL. Binary regression using an extended beta-binomial distribution, with discussion of correlation induced by covariate measurement errors. J Am Stat Assoc. 1986;81(394):321–7.

5 Gelman A, Carlin JB, Stern HS, Dunson DB, Rubin DB: Bayesian data analysis, third edition. Journal of the American Statistical Association. 2003;45(2):.
