# Supplementary material for: Transmission risks of Omicron BA.5 following inactivated COVID-19 vaccines among children and adolescents in China
Source: Commun Med (Lond). 2024 May 18;4:92. doi: 10.1038/s43856-024-00521-y (PMC11102477; doi:10.1038/s43856-024-00521-y)
Supplement: Supplementary file 2 — Description of Additional Supplementary Files [file 43856_2024_521_MOESM2_ESM.pdf]

Description of Additional Supplementary Files

File name: Supplementary Data 1

Description: The estimated effectiveness of inactivated COVID-19 vaccines (mainly BBIBP-CorV) in preventing the risk of Omicron BA.5 infection (regardless of symptoms), symptomatic infection only, and the transmission risk of Omicron BA.5 in children and adolescents.

File name: Supplementary

Description:

File name: Supplementary

Description:

File name: Supplementary

Description:
